# Endoscopic tri-modal imaging for surveillance in ulcerative colitis: randomised comparison of high-resolution endoscopy and autofluorescence imaging for neoplasia detection; and evaluation of narrow-band imaging for classification of lesions

**DOI:** 10.1136/gut.2007.144097

**Published:** 2008-03-26

**Authors:** F J C van den Broek, P Fockens, S van Eeden, J B Reitsma, J C H Hardwick, P C F Stokkers, E Dekker

**Affiliations:** 1 Department of Gastroenterology and Hepatology, Academic Medical Centre Amsterdam, Netherlands; 2 Pathology, Academic Medical Centre Amsterdam, Netherlands; 3 Clinical Epidemiology, Biostatistics and Bioinformatics, Academic Medical Centre Amsterdam, Netherlands

## Abstract

**Background:**

Endoscopic tri-modal imaging (ETMI) incorporates white light endoscopy (WLE), autofluorescence imaging (AFI) and narrow-band imaging (NBI).

**Aims:**

To assess the value of ETMI for the detection and classification of neoplasia in patients with longstanding ulcerative colitis.

**Design:**

Randomised comparative trial of tandem colonoscopies.

**Setting:**

Academic Medical Centre Amsterdam, Netherlands.

**Patients and methods:**

Fifty patients with ulcerative colitis underwent surveillance colonoscopy with ETMI. Each colonic segment was inspected twice, once with AFI and once with WLE, in random order. All detected lesions were inspected by NBI for Kudo pit pattern analysis and additional random biopsies were taken.

**Main outcome measures:**

Neoplasia miss-rates of AFI and WLE, and accuracy of the Kudo classification by NBI.

**Results:**

Among patients assigned to inspection with AFI first (n = 25), 10 neoplastic lesions were primarily detected. Subsequent WLE detected no additional neoplasia. Among patients examined with WLE first (n = 25), three neoplastic lesions were detected; subsequent inspection with AFI added three neoplastic lesions. Neoplasia miss-rates for AFI and WLE were 0% and 50% (p = 0.036). The Kudo classification by NBI had a sensitivity and specificity of 75% and 81%; however, all neoplasia was coloured purple on AFI (sensitivity 100%). No additional patients with neoplasia were detected by random biopsies.

**Conclusion:**

Autofluorescence imaging improves the detection of neoplasia in patients with ulcerative colitis and decreases the yield of random biopsies. Pit pattern analysis by NBI has a moderate accuracy for the prediction of histology, whereas AFI colour appears valuable in excluding the presence of neoplasia.

**Trial registration number:**

ISRCTN05272746

Patients with longstanding ulcerative colitis are at increased risk of developing colorectal cancer.^1^ ^2^ Since colonoscopic surveillance in ulcerative colitis patients appears to lead to early detection and improved prognosis of neoplasia, guidelines recommend surveillance for these patients.^3^^–^6 However, neoplasia mainly develops in flat mucosa, so it can easily be overlooked during colonoscopy.^7^^–^10 Therefore, random biopsies are recommended in addition to targeted biopsies of suspicious lesions.^4^^–^6 Despite these great efforts, neoplasia is still frequently being missed by colonoscopy, possibly leading to interval cancers.^11^ ^12^

New endoscopic imaging techniques aim to facilitate the detection of neoplasia.^13^ Chromoendoscopy (CE) has been proven to increase the detection of neoplasia in ulcerative colitis and, additionally, enables pit pattern analysis for an accurate endoscopic classification by experts.^14^^–^17 Nevertheless, implementation of CE in clinical practice has fallen short since it is labour-intensive and requires special training. By contrast, narrow-band imaging (NBI) utilises spectral characteristics of endoscopic light to enhance mucosal details without dyes.^18^^–^20 Recently, NBI failed to improve the detection of neoplasia in ulcerative colitis,^11^ yet has been judged a valuable tool for classification.^21^ ^22^ Whereas the diagnostic accuracy of NBI for differentiation of neoplastic and non-neoplastic tissue in ulcerative colitis remains to be clarified, its accuracy for differentiating sporadic polyps has shown to be comparable to CE.^23^^–^27

Autofluorescence imaging (AFI) is another novel technique, using short-wavelength (blue) light for excitation of endogenous tissue fluorophores which emit fluorescent light of longer wavelength.^28^ Therefore, AFI highlights neoplastic tissue without administration of exogenous fluorophores as described before in ulcerative colitis patients.^29^ ^30^

Recently, high-resolution white-light endoscopy (WLE), AFI and NBI have been incorporated into one system: endoscopic tri-modal imaging (ETMI).^31^ To date, ETMI has only been evaluated in patients with Barrett’s oesophagus and AFI has only been described in two patients with ulcerative colitis.^32^ ^33^ The aims of this randomised study were to compare AFI and WLE for the detection of neoplasia in patients with longstanding ulcerative colitis, and to assess the accuracy of NBI for pit pattern classification.

## PATIENTS AND METHODS

### Participants

Patients with inactive pan-ulcerative colitis ⩾8 years scheduled for surveillance colonoscopy at the Academic Medical Centre Amsterdam were invited for this study. A modified Truelove & Witts severity index ⩽2 was used to define inactive disease.^34^ Exclusion criteria were insufficient bowel preparation, endoscopically active inflammation, age ⩽18 years, non-correctable coagulopathy, and inability to give informed consent.

### Endoscopy equipment

**Figure 1:**
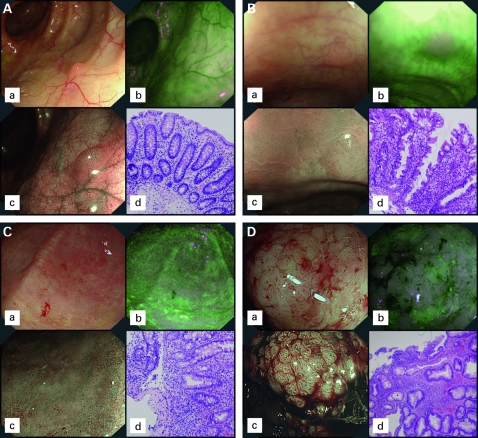
(A) Images during high-resolution white-light endoscopy (WLE) (a); autofluorescence imaging (AFI) (b); and narrow-band imaging (NBI) (c), of mucosa with (d) no significant changes on histology. On AFI normal mucosa appears green; NBI shows a normal pit pattern (Kudo type I). (B). Images during WLE (a), AFI (b), and NBI (c), of a lesion revealing hyperplastic-like mucosal changes on histopathology (d). Tissue autofluorescence is disturbed leading to a purple (false positive) colour on AFI; during NBI a normal pit pattern is seen. (C) Images during WLE (a), AFI (b), and NBI (c), of an area showing inflammation on histopathology (d). On AFI, inflammation becomes purple (false positive), drawing the attention of the endoscopist. On NBI, an irregular pit pattern is seen, partly with elongated pits (Kudo type IIIL). (D) Images during WLE (a), AFI (b), and NBI (c), of a mass revealing low-grade intraepithelial neoplasia on histopathology (d). The neoplastic lesion appears deep purple on AFI and reveals Kudo pit pattern type IV on NBI.

All colonoscopies were performed with a prototype ETMI system (Olympus Inc., Tokyo, Japan). The light source (XCLV-260HP) contains two rotating red–green–blue RGB filters; one conventional for WLE and one additional for NBI, in which the band-pass ranges are narrowed to wavelengths of 530–550 nm (green) and 390–445 nm (blue). For NBI, the relative intensity of blue light is increased. Since blue light penetrates the mucosa only superficially and is the main colour absorbed by haemoglobin, this setting enhances surface and capillary details (fig 1A–D).^18^ ^19^

The zoom video-colonoscope (XCF-H240FZL; magnification ×100) contains two charge-coupled devices, one for WLE/NBI and one for AFI. In the AFI mode, blue light (390–470 nm) is used for excitation and green light (540–560 nm) for reflection. A barrier filter is used to detect autofluorescence and reflected light with wavelengths of 500–630 nm only. The sequentially detected images of autofluorescence and green reflection are integrated by the processor into a real-time pseudo-colour image. During AFI normal mucosa appears green, while neoplasia appears purple (fig 1A,D).

A high-resolution monitor was used for all procedures, in which the endoscopist could easily switch between the three imaging modes by pressing a button on the shaft of the endoscope.

### Colonoscopic procedure and randomisation

Patients were prepared with 4 litres of hypertonic polyethylene glycol solution (Kleanprep; Norgine, Marburg, Germany) and received conscious sedation. The endoscope was advanced in the WLE mode and caecal intubation was confirmed by identification of the appendiceal orifice and ileocaecal valve. No biopsies were taken during insertion of the endoscope. All procedures were performed by three experienced colonoscopists (>2500 colonoscopies).

Upon reaching the caecum, the level of bowel preparation was determined: good (100% visible mucosa), moderate (90–100%) or poor (<90%). On introduction of the endoscope efforts were made to optimally clean the bowel by rinsing and suctioning. Poor bowel preparation was an exclusion criterion, as well as endoscopically active disease in at least one colonic segment.

**Figure 2:**
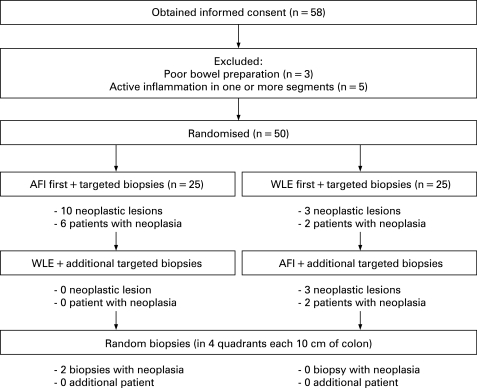
Study design and flow chart of patients who gave informed consent during the study. The number of detected neoplastic lesions and number of patients with neoplasia are summarised per randomisation group. During white-light endoscopy (WLE), three neoplastic lesions (50%) were missed which were detected by autofluorescence imaging (AFI). Two random biopsies showed neoplasia after inspection with AFI and subsequent WLE; these were found in a patient in whom AFI already detected three areas of flat neoplasia.

During withdrawal of the colonoscope, each colonic segment was inspected twice: once with AFI and once with WLE. The hepatic and splenic flexures separated the colonic segments; in case of indistinct flexures a biopsy was taken for reference during the second inspection. Patients were randomly allocated for inspection with AFI or WLE first in all segments (fig 2). One hundred opaque sealed envelopes contained notes with “AFI” or “WLE” written on them (1:1) for randomisation. At the moment of caecal intubation, a research fellow opened a randomly chosen envelope for allocation. The endoscopists were instructed to have equal inspection times for AFI and WLE. In a random sample of 10 patients inspection times were measured for both AFI and WLE, strictly excluding time for rinsing, taking pictures and taking biopsies. Suspicious lesions detected during the first inspection were sampled immediately after detection. During the second inspection, only additionally detected suspicious lesions were sampled. Finally, four quadrant random biopsies were taken every 10 cm of colon. For each lesion, the detection technique (WLE or AFI), size (estimated by a biopsy forceps), Paris classification, and location (part of colon and distance from the anus) were noted.^35^ Furthermore, AFI colour (green, ambiguous or purple) was scored as well as the Kudo classification by NBI and magnification.^36^

During inspection with WLE, suspicious lesions were defined as polypoid or irregular mucosal structures, unusual ulcers and strictures. During AFI, all areas with ambiguous/purple colour were considered suspicious.

### Histopathological diagnosis

Biopsies were evaluated by two blinded pathologists, one of them considered a gastrointestinal expert. Biopsies were classified according to the Vienna criteria of gastrointestinal epithelial neoplasia.^37^ In the case of discrepancy between the pathologists, discussion led to a consensus diagnosis. Low-grade (LGIN) and high-grade intraepithelial neoplasia (HGIN), as well as invasive neoplasia were defined as neoplasia; lesions diagnosed as indefinite for neoplasia were not considered neoplastic.

A significant number of biopsies showed hypermucinous, serrated, epithelial changes reminiscent of hyperplastic polyps, which could not be recognised as overt intraepithelial neoplasia and of which the significance is unknown. These lesions appear similar to the lesions described by Kilgore *et al*^38^ in Crohn’s disease as hyperplastic-like mucosal changes (HPC). The presence of these lesions was recorded separately and the term “HPC” was adopted from the study by Kilgore *et al*.

### Primary and secondary outcomes

The primary outcomes were the proportions of neoplastic lesions missed by AFI or WLE, and the accuracy of the Kudo classification assessed by NBI. Secondary outcomes were proportions of patients with missed neoplasia, patients with neoplasia detected by random biopsies only, number of false positive lesions and the accuracy of AFI colour.

### Statistical methods

Continuous data were represented by their mean±standard deviation (SD) or by their median±interquartile range (IQR) when appropriate. Differences were tested by the Student t test or Wilcoxon rank test, respectively. Proportions were compared by the χ^2^ or Fisher’s exact test.

Since lesions were sampled immediately after detection, only missed lesions could be detected by the second technique, which precludes a paired analysis. In order to compare AFI and WLE for neoplasia detection, we therefore compared the number of neoplastic lesions detected by the second inspection divided by the total number of detected neoplastic lesions (first and second inspection), representing the miss-rate for each technique. These proportions were compared using Fisher’s exact test. The proportions of patients with missed neoplasia were compared in the same manner.

Contingency tables were used to determine sensitivity, specificity and overall accuracy (95% confidence intervals) of the Kudo classification by NBI and of the AFI colour. Histopathology was used as the “gold standard”.

The number of false positive lesions during AFI and WLE (suspicious lesion on endoscopy but negative for neoplasia on histology) was compared using the paired Wilcoxon rank test.

### Sample size

When previously applying identical inclusion criteria, the neoplasia prevalence was 29% and the neoplasia miss-rate for WLE was 54% (per lesion analysis).^11^ We assumed the neoplasia miss-rate for AFI to be 10% and expected patients with neoplasia to have an average of 2.5 lesions. This resulted in a sample size of 50 patients (α-error 0.05 and β-error 0.2).

## RESULTS

### Patient characteristics

From April 2005 to November 2006 a total of 58 patients gave informed consent. Eight patients were excluded because of poor bowel preparation (n = 3) or endoscopically active disease (n = 5). Therefore, 50 patients were randomised for tandem colonoscopy; in 25 patients the first inspection was done with AFI, in the other 25 patients WLE was used first (fig 2). Baseline characteristics are summarised according to randomisation in table 1. The three endoscopists performed 18, 18 and 14 procedures each and no complications occurred in any of the patients.

**Table 1:** Baseline patient characteristics among patients randomly assigned to autofluorescence imaging (AFI) and white-light endoscopy (WLE) as the first examination technique

Characteristic	Randomisation		p Value
	AFI first (n = 25)	WLE first (n = 25)	
Male, n (%)	17 (68%)	14 (56%)	0.382
Mean age, years (SD)	50 (11)	51 (13)	0.889
Duration of median ulcerative colitis, years (IQR)	16 (12–21)	14 (12–20)	0.651
History of neoplasia, n (%)	3 (12%)	4 (16%)	1.0
Primary sclerosing cholangitis, n (%)	3 (12%)	4 (16%)	1.0
Disease-modifying drug use, n (%)	23 (92%)	18 (72%)	0.138
Good colon preparation, n (%)	14 (56%)	20 (80%)	0.069

### Duration of colonoscopy and number of suspicious lesions

The mean inspection time for AFI was 8.1 (SD 2.7) min and for WLE 7.9 (SD 3.9) min (p = 0.757). During examination with AFI first, 37 suspicious lesions were detected (16 patients); second inspection with WLE added seven lesions (seven patients). Inspection with WLE first yielded 34 suspicious lesions (18 patients) and subsequent AFI added 20 lesions (11 patients).

### Neoplasia in targeted biopsies

#### Autofluorescence imaging first

Among the 25 patients assigned to inspection with AFI first, 10 neoplastic lesions were detected with AFI (six patients). Subsequent examination with WLE detected no additional neoplasia (fig 2). No lesions indefinite for neoplasia were found in this group.

The first patient had three flat elevated lesions of 4–10 cm throughout the colon, all harbouring LGIN; subsequent colectomy demonstrated LGIN and focal HGIN at the same colonic sites as during colonoscopy. The second patient also had three flat elevated lesions, all 4 mm in size revealing LGIN. Given the small size, these lesions were considered as adenoma-like masses (ALMs) and removed by endoscopic mucosal resection; repeat colonoscopy within 1 year again revealed LGIN. Three other patients harboured one flat lesion each (of 3, 5 and 17 mm) with LGIN, which were considered as ALMs although one of these patients had three areas of flat LGIN detected within 1 year. The last patient harboured a 10 cm irregular area with focal HGIN which had already been noticed on introduction of the endoscope. This patient underwent colectomy in which no neoplasia could be demonstrated; after reviewing the original biopsies there was no doubt about the initial diagnosis of HGIN.

#### White-light endoscopy first

Among 25 patients assigned to WLE first, three neoplastic lesions were detected with WLE (two patients). Subsequent inspection with AFI added three neoplastic lesions (two patients). No lesions indefinite for neoplasia were found.

The first patient with WLE detected neoplasia had a 6 mm flat elevated lesion with LGIN, considered as ALM. The second patient harboured a 6 cm nodular flat lesion in the caecum and a 1 cm polypoid lesion in the descending colon, both revealing LGIN. Total colectomy in that patient only confirmed LGIN in the caecum. All neoplastic lesions detected by WLE were coloured purple on AFI. White-light endoscopy failed to detect three lesions with LGIN in two patients, which were detected by AFI during the second inspection. In one patient, two sessile lesions of 3 mm were missed in the sigmoid colon revealing LGIN; in the other patient a sessile lesion of 5 mm was missed revealing LGIN. Repeat colonoscopy within 1 year again revealed LGIN at other areas in both patients with missed neoplasia.

Of the 10 patients with neoplasia, three were referred to our hospital to confirm the presence of neoplasia, three had a history of ALM resection at our own institution and one had previous neoplasia in random biopsies. Only one patient with neoplasia had a history of primary sclerosing cholangitis (PSC). There were no differences in neoplasia detection between endoscopists or between early or late ETMI procedures.

### Neoplasia in random biopsies

In total, 1992 random biopsies were taken among 50 patients. In two biopsies (0.1%) histopathology revealed LGIN, both taken in a patient in whom AFI already demonstrated three neoplastic lesions, confirmed by colectomy. The first positive random biopsy was taken in an AFI purple region of which targeted biopsies revealed inflammation on histology; the second was taken just proximal to a neoplasia detected by AFI. Furthermore, eight random biopsies showed mucosal changes indefinite for neoplasia (four patients), 252 inflammation, 100 HPC and 1632 showed no significant changes.

Two of the four patients with indefinite neoplasia in random biopsies underwent colectomy for neoplasia in targeted biopsies as well; in the remaining two patients with indefinite neoplasia, three subsequent follow-up colonoscopies did not reveal any neoplasia.

### Neoplasia miss-rates

The percentage of missed neoplastic lesions was 0% (0/10) for AFI and 50% (3/6) for WLE (p = 0.036). The percentage of patients with neoplasia missed by AFI was 0% (0/6) vs 50% (2/4) for WLE (p = 0.133).

When including neoplasia detected by random biopsies (n = 2), corresponding neoplasia miss-rates for AFI and WLE were 17% (2/12) and 50% (3/6) respectively (p = 0.268).

### False positive findings

During inspection with AFI, a total of 44 false positive lesions were found compared to 38 during WLE (p = 0.882). Of all false positive lesions by AFI, histology showed inflammation in 22 lesions (50%) and HPC in two (4.5%). Histology of false positive lesions by WLE showed inflammation in six lesions (16%) and HPC in 13 (34%). All other false positive lesions showed no significant changes on histology.

### Findings on narrow-band imaging and autofluorescence imaging compared to histology

Fifty-seven suspicious lesions were found with AFI and 41 with WLE, which were all inspected by NBI before taking biopsies. Of the 16 histologically proven neoplastic lesions, four showed non-neoplastic pit patterns (type I–II) on NBI; of the 82 remaining non-neoplastic lesions, 16 demonstrated neoplastic pit patterns (type III–V) due to chronic inflammation. The sensitivity, specificity and overall accuracy of the Kudo classification by NBI were 75% (95% CI, 51 to 90), 81% (71 to 88) and 80% (71 to 86) respectively (table 2).

**Table 2:** Correspondence between the Kudo pit pattern classification (by narrow-band imaging (NBI)) and histopathology (the gold standard) of all detected lesions

NBI classification	Histopathology		Total
	Neoplastic	Non-neoplastic	
Suspicious	12	16	28, PPV 43%
Non-suspicious	4	66	70, NPV 94%
Total	16,sens. 75%	82,spec. 81%	98

Kudo pit pattern type I–II was considered as non-suspicious and Kudo type III–V as suspicious for neoplasia.

NPV, negative predictive value; PPV, positive predictive value; sens., sensitivity; spec., specificity.

All suspicious lesions were also scored for colour on AFI. Considering AFI green as non-neoplastic and AFI purple/ambiguous as neoplastic the sensitivity, specificity and overall accuracy of AFI colour were 100% (81 to 100), 42% (31 to 52) and 51% (41 to 61), respectively.

When combining AFI and NBI findings, thereby considering AFI green as well as all AFI-ambiguous lesions with Kudo type I–II on NBI as negative for neoplasia, the sensitivity, specificity and overall accuracy were 100% (81 to 100), 72% (61 to 81) and 77% (67 to 84). This combined use of AFI plus NBI could hypothetically have prevented taking targeted biopsies of 59 lesions (60%) without leaving neoplasia in situ.

## DISCUSSION

This is the first randomised study comparing AFI to WLE for detection and NBI for classification of neoplasia in patients with longstanding ulcerative colitis. We have demonstrated that ETMI is feasible for colonic use, provided that the colon is properly prepared and not actively inflamed. Insufficient bowel preparation and active inflammation interrupt tissue autofluorescence, resulting in discoloration on AFI and resembling neoplasia.^39^^–^42 Active inflammation is an exclusion criterion for surveillance in any ulcerative colitis patient, since histopathological distinction between inflammation and neoplasia can be extremely difficult or even impossible in this situation.^43^ ^44^ Disrupted autofluorescence due to residual faeces may explain why patients allocated to AFI inspection first were less often judged to have good colon preparation as shown in table 1 (p = 0.069). However, insufficient bowel preparation does not uniquely preclude scrutinising the colon during AFI, but this is true for WLE as well. When becoming accustomed to AFI during the study period, the percentage colon preparation judged as good increased from 22% to 75% (early *v* late procedures; p = 0.017) among patients allocated to AFI first.

The present study demonstrated a neoplasia miss-rate of 50% for WLE compared to a miss-rate of 0% for AFI (p = 0.036). Although all missed neoplastic lesions were considered ALMs and have been treated by endoscopic mucosal resection, repeat colonoscopy within 1 year confirmed the presence of neoplasia in these patients. Debate still exists about whether ALMs should prompt colectomy or can safely be removed by endoscopic resection.^45^ An important consideration is whether ALMs are located within or proximal to the extent of inflamed colon. All included patients had pancolitis and therefore hold a higher risk for cancer development, even if neoplasia was considered as an ALM. Since only intraepithelial neoplasias have been found in our study, the value of AFI for the detection of early invasive cancers could not be evaluated.

We found a high prevalence of neoplasia in our study population, probably caused by the tertiary referral function of our institution, selection of patients with pancolitis, and inclusion of patients with a history of neoplasia without colectomy. This high prevalence at our institution has been demonstrated before in a study comparing standard WLE and NBI, revealing a neoplasia miss-rate of 42% for WLE.^11^ The even higher miss-rate for high resolution WLE in the present study suggests that AFI may correctly visualise a significant part of neoplasia, which remains invisible for WLE. Despite appropriate powering of the present study, however, the relatively small sample size prompts confirmation of these results in larger trials.

In a retrospective study, standard colonoscopy missed 39% of all neoplasia which was only detected by additional random biopsies.^10^ The authors included neoplasia detected by random biopsies in the denominator for measuring sensitivity and no additional imaging techniques were used, as opposed to our study. The neoplasia miss-rate for high resolution WLE in our study was 50%, only missing lesions that were subsequently visualised by AFI. Random biopsies did not add neoplasia in any of these cases. Among patients examined with AFI first and WLE second, two random biopsies revealed neoplasia, which may be analysed as neoplasia missed by both AFI and WLE. When including positive random biopsies, the neoplasia miss-rate of AFI was 17% (2/12), which is still lower than current practice. Two remarks must be made concerning those random biopsies revealing “invisible” neoplasia. First, the positive biopsies were both derived from one patient in whom AFI already demonstrated three “visible” neoplastic lesions. Second, one out of two biopsies was taken in an AFI purple region and one was taken adjacent to a neoplasia visualised by AFI. The presence of neoplasia in biopsies taken adjacent to visible neoplasia merely reflects the nature of dysplasia associated lesions or masses instead of missed neoplasia.^46^ Therefore, we conclude that random biopsies did not add relevant neoplasia.

The need for random biopsies in addition to the use of advanced imaging techniques has been questioned several times.^11^ ^15^ ^47^ Random biopsies have a low yield of neoplasia when used next to CE.^16^ Also, in the present study the diagnostic yield of random biopsies was low, only finding neoplasia in 0.1% of biopsies. Random biopsies increase examination time and pathology costs, may distract the endoscopist from scrutinising the colon and have a risk of bleeding. The fact that all neoplasia in this study was coloured purple on AFI and that random biopsies did not detect neoplasia in additional patients, underlines the question whether these biopsies should be taken if AFI reveals a colon with the normal “green” appearance.

The high yield of neoplasia with AFI must be weighed against the results of CE in previous studies.^14^^–^17 In our experience, the use of AFI is easier and more convenient for both patients and endoscopists. Only pressing a button instead of applying dyes to the mucosa will provide enhanced imaging, thereby saving time. We experienced AFI to have a lower resolution compared to high-resolution WLE, although it served well as a red flag technique. The need for high resolution might be questioned in the circumstance where AFI correctly visualised all neoplasia by colouring purple. The results of the present study support the use of AFI, but the small sample size and expert setting prompt larger trials comparing AFI and CE in general practice to provide practical recommendations.

Although AFI has demonstrated a high rate of false positive findings in Barrett’s oesophagus, the present study showed that there was no difference in false positive rate among AFI or WLE in ulcerative colitis surveillance.^32^ ^41^ ^48^ As known from previous research, active inflammation colours purple during AFI.^42^ The present study demonstrated purple coloration of chronic inflammation as well, which was the main cause of false positive finding for AFI, whereas hyperplastic-like mucosal change (HPC) was the most notable false positive finding for WLE. These HPCs might be similar to the lesions described before in Crohn’s disease, in which an association was shown between the prevalence of HPC and concomitant neoplasia.^38^ We could not demonstrate a correlation between HPC and neoplasia in our study (results not shown), although prospective evaluation and molecular analysis of these lesions might be an interesting subject for future studies.

Confocal endomicroscopy has recently proven to be a good candidate to reduce false positive biopsies and to increase the effectiveness of surveillance, although this technique requires expensive equipment and special training.^15^ ^49^ In the present study the Kudo classification with NBI had an unsatisfactory specificity and sensitivity of 81% and 75%, figures which are comparable to a recent report on pit pattern analysis with CE.^49^ On the contrary, all neoplasias were coloured purple on AFI. Therefore, the sensitivity of NBI could be improved by adding the information about AFI colour in the rule for defining a positive test result. All AFI green lesions and all AFI ambiguous lesions with Kudo type I–II on NBI revealed non-neoplastic histology, suggesting that those lesions can safely be left in situ. Prospective studies should validate this finding, which might lead to a sensitivity of 100% and specificity of 77%.

Instead of Kudo pit pattern analysis with NBI, the vascular intensity pattern has also been used for differentiation of lesions in the non-inflamed colon.^24^^–^26 In a recent study by East *et al*^27^ the accuracy of the Kudo classification was comparable to the vascular intensity pattern in the non-inflamed colon. However, the value of vascular pattern analysis in patients with ulcerative colitis is still unknown.

In summary, ETMI appears a feasible option for colonoscopic surveillance of patients with ulcerative colitis, although additional studies are imperative. In the present trial AFI reduced the neoplasia miss-rate to 0% and made random biopsies superfluous. For the endoscopic classification of lesions, NBI had a sensitivity of only 75% but the additional information about colour obtained by AFI increased the sensitivity to 100%. Larger studies in a non-expert setting are needed to confirm these results, as well as comparative trials with chromoendoscopy and confocal endomicroscopy in order to demonstrate which technique performs better and is more convenient for the detection and classification of neoplasia.

## References

[1] Itzkowitz SH Harpaz N Diagnosis and management of dysplasia in patients with inflammatory bowel diseases. Gastroenterology 2004;126:1634–48 15168373 10.1053/j.gastro.2004.03.025

[2] Eaden JA Abrams KR Mayberry JF The risk of colorectal cancer in ulcerative colitis: a meta-analysis. Gut 2001;48:526–35 11247898 10.1136/gut.48.4.526 PMC1728259

[3] Collins P Mpofu C Watson A Strategies for detecting colon cancer and/or dysplasia in patients with inflammatory bowel disease. Cochrane Database Syst Rev 2006;(2):CD000279 16625534 10.1002/14651858.CD000279.pub3

[4] Itzkowitz SH Present DH Consensus conference: Colorectal cancer screening and surveillance in inflammatory bowel disease. Inflamm Bowel Dis 2005;11:314–21 15735438 10.1097/01.mib.0000160811.76729.d5

[5] Eaden JA Mayberry JF Guidelines for screening and surveillance of asymptomatic colorectal cancer in patients with inflammatory bowel disease. Gut 2002;51 (Suppl 5):V10–V12 12221032 10.1136/gut.51.suppl_5.v10 PMC1867735

[6] Labianca R Beretta GD Mosconi S Colorectal cancer: screening. Ann Oncol 2005;16 (Suppl 2):ii127–ii132 15958442 10.1093/annonc/mdi730

[7] Ransohoff DF Riddell RH Levin B Ulcerative colitis and colonic cancer. Problems in assessing the diagnostic usefulness of mucosal dysplasia. Dis Colon Rectum 1985;28:383–8 4006632 10.1007/BF02560215

[8] Tytgat GN Dhir V Gopinath N Endoscopic appearance of dysplasia and cancer in inflammatory bowel disease. Eur J Cancer 1995;31A:1174–7 7577016 10.1016/0959-8049(95)00133-4

[9] Rutter M Bernstein C Matsumoto T Endoscopic appearance of dysplasia in ulcerative colitis and the role of staining. Endoscopy 2004;36:1109–14 15578305 10.1055/s-2004-826049

[10] Rubin DT Rothe JA Hetzel JT Are dysplasia and colorectal cancer endoscopically visible in patients with ulcerative colitis? Gastrointest Endosc 2007;65:998–1004 17451704 10.1016/j.gie.2006.09.025

[11] Dekker E van den Broek FJ Reitsma JB Narrow-band imaging compared with conventional colonoscopy for the detection of dysplasia in patients with longstanding ulcerative colitis. Endoscopy 2007;39:216–21 17385106 10.1055/s-2007-966214

[12] Rutter MD Saunders BP Wilkinson KH Thirty-year analysis of a colonoscopic surveillance program for neoplasia in ulcerative colitis. Gastroenterology 2006;130:1030–8 16618396 10.1053/j.gastro.2005.12.035

[13] Dekker E Fockens P New imaging techniques at colonoscopy: tissue spectroscopy and narrow band imaging. Gastrointest Endosc Clin N Am 2005;15:703–14 16278134 10.1016/j.giec.2005.08.006

[14] Kiesslich R Fritsch J Holtmann M Methylene blue-aided chromoendoscopy for the detection of intraepithelial neoplasia and colon cancer in ulcerative colitis. Gastroenterology 2003;124:880–8 12671882 10.1053/gast.2003.50146

[15] Kiesslich R Goetz M Lammersdorf K Chromoscopy-guided endomicroscopy increases the diagnostic yield of intraepithelial neoplasia in ulcerative colitis. Gastroenterology 2007;132:874–82 17383417 10.1053/j.gastro.2007.01.048

[16] Rutter MD Saunders BP Schofield G Pancolonic indigo carmine dye spraying for the detection of dysplasia in ulcerative colitis. Gut 2004;53:256–60 14724160 10.1136/gut.2003.016386 PMC1774934

[17] Hurlstone DP Sanders DS Lobo AJ Indigo carmine-assisted high-magnification chromoscopic colonoscopy for the detection and characterisation of intraepithelial neoplasia in ulcerative colitis: a prospective evaluation. Endoscopy 2005;37:1186–92 16329015 10.1055/s-2005-921032

[18] Gono K Obi T Yamaguchi M Appearance of enhanced tissue features in narrow-band endoscopic imaging. J Biomed Opt 2004;9:568–77 15189095 10.1117/1.1695563

[19] Kuznetsov K Lambert R Rey JF Narrow-band imaging: potential and limitations. Endoscopy 2006;38:76–81 16429359 10.1055/s-2005-921114

[20] Gheorghe C Narrow-band imaging endoscopy for diagnosis of malignant and premalignant gastrointestinal lesions. J Gastrointestin Liver Dis 2006;15:77–82 16680239

[21] East JE Suzuki N von HA Narrow band imaging with magnification for dysplasia detection and pit pattern assessment in ulcerative colitis surveillance: a case with multiple dysplasia associated lesions or masses. Gut 2006;55:1432–5 16966701 10.1136/gut.2005.087171 PMC1856415

[22] Matsumoto T Kudo T Jo Y Magnifying colonoscopy with narrow band imaging system for the diagnosis of dysplasia in ulcerative colitis: a pilot study. Gastrointest Endosc 2007;66:957–65 17826773 10.1016/j.gie.2007.04.014

[23] Machida H Sano Y Hamamoto Y Narrow-band imaging in the diagnosis of colorectal mucosal lesions: a pilot study. Endoscopy 2004;36:1094–8 15578301 10.1055/s-2004-826040

[24] Su MY Hsu CM Ho YP Comparative study of conventional colonoscopy, chromoendoscopy, and narrow-band imaging systems in differential diagnosis of neoplastic and nonneoplastic colonic polyps. Am J Gastroenterol 2006;101:2711–6 17227517 10.1111/j.1572-0241.2006.00932.x

[25] Chiu HM Chang CY Chen CC A prospective comparative study of narrow-band imaging, chromoendoscopy, and conventional colonoscopy in the diagnosis of colorectal neoplasia. Gut 2006;56:373–9 17005766 10.1136/gut.2006.099614 PMC1856788

[26] Hirata M Tanaka S Oka S Magnifying endoscopy with narrow band imaging for diagnosis of colorectal tumors. Gastrointest Endosc 2007;65:988–95 17324407 10.1016/j.gie.2006.07.046

[27] East JE Suzuki N Saunders BP Comparison of magnified pit pattern interpretation with narrow band imaging versus chromoendoscopy for diminutive colonic polyps: a pilot study. Gastrointest Endosc 2007;66:310–6 17643705 10.1016/j.gie.2007.02.026

[28] DaCosta RS Wilson BC Marcon NE Optical techniques for the endoscopic detection of dysplastic colonic lesions. Curr Opin Gastroenterol 2005;21:70–9 15687888

[29] Messmann H Endlicher E Freunek G Fluorescence endoscopy for the detection of low and high grade dysplasia in ulcerative colitis using systemic or local 5-aminolaevulinic acid sensitisation. Gut 2003;52:1003–7 12801958 10.1136/gut.52.7.1003 PMC1773731

[30] Ochsenkuhn T Tillack C Stepp H Low frequency of colorectal dysplasia in patients with long-standing inflammatory bowel disease colitis: detection by fluorescence endoscopy. Endoscopy 2006;38:477–82 16767582 10.1055/s-2006-925165

[31] Kara MA Bergman JJ Autofluorescence imaging and narrow-band imaging for the detection of early neoplasia in patients with Barrett’s esophagus. Endoscopy 2006;38:627–31 16802271 10.1055/s-2006-925385

[32] Curvers WL Singh R Song LM Endoscopic tri-modal imaging for detection of early neoplasia in Barrett’s oesophagus: a multi-centre feasibility study using high-resolution endoscopy, autofluorescence imaging and narrow band imaging incorporated in one endoscopy system. Gut 2008;57:167–72 17965067 10.1136/gut.2007.134213

[33] Matsumoto T Moriyama T Yao T Autofluorescence imaging colonoscopy for the diagnosis of dysplasia in ulcerative colitis. Inflamm Bowel Dis 2007;13:640–1 17221857 10.1002/ibd.20104

[34] Lichtiger S Present DH Kornbluth A Cyclosporine in severe ulcerative colitis refractory to steroid therapy. N Engl J Med 1994;330:1841–5 8196726 10.1056/NEJM199406303302601

[35] Endoscopic Classification Review Group Update on the paris classification of superficial neoplastic lesions in the digestive tract. Endoscopy 2005;37:570–8 15933932 10.1055/s-2005-861352

[36] Kudo S Rubio CA Teixeira CR Pit pattern in colorectal neoplasia: endoscopic magnifying view. Endoscopy 2001;33:367–73 11315901 10.1055/s-2004-826104

[37] Schlemper RJ Riddell RH Kato Y The Vienna classification of gastrointestinal epithelial neoplasia. Gut 2000;47:251–5 10896917 10.1136/gut.47.2.251 PMC1728018

[38] Kilgore SP Sigel JE Goldblum JR Hyperplastic-like mucosal change in Crohn’s disease: an unusual form of dysplasia? Mod Pathol 2000;13:797–801 10912940 10.1038/modpathol.3880138

[39] Brand S Stepp H Ochsenkuhn T Detection of colonic dysplasia by light-induced fluorescence endoscopy: a pilot study. Int J Colorectal Dis 1999;14:63–8 10207734 10.1007/s003840050186

[40] Haringsma J Tytgat GN Yano H Autofluorescence endoscopy: feasibility of detection of GI neoplasms unapparent to white light endoscopy with an evolving technology. Gastrointest Endosc 2001;53:642–50 11323596 10.1067/mge.2001.114419

[41] Kara MA Peters FP ten Kate FJ Endoscopic video autofluorescence imaging may improve the detection of early neoplasia in patients with Barrett’s esophagus. Gastrointest Endosc 2005;61:679–85 15855971 10.1016/s0016-5107(04)02577-5

[42] Matsumoto T Kudo T Yao T Autofluorescence imaging colonoscopy in ulcerative colitis: Comparison with conventional and narrow-band imaging colonoscopy. Dig Endosc 2007;19 (Suppl 1):S139–S144 10.1111/j.1443-1661.2011.01110.x 21535220

[43] Riddell RH Goldman H Ransohoff DF Dysplasia in inflammatory bowel disease: standardized classification with provisional clinical applications. Hum Pathol 1983;14:931–68 6629368 10.1016/s0046-8177(83)80175-0

[44] Rubin DT Turner JR Surveillance of dysplasia in inflammatory bowel disease: The gastroenterologist–pathologist partnership. Clin Gastroenterol Hepatol 2006;4:1309–13 17110299 10.1016/j.cgh.2006.09.010 PMC1829445

[45] Bernstein CN ALMs versus DALMs in ulcerative colitis: polypectomy or colectomy? Gastroenterology 1999;117:1488–92 10579991 10.1016/s0016-5085(99)70300-8

[46] Hurlstone DP Sanders DS Atkinson R Endoscopic mucosal resection for flat neoplasia in chronic ulcerative colitis: can we change the endoscopic management paradigm? Gut 2007;56:838–46 17135310 10.1136/gut.2006.106294 PMC1954845

[47] Rutter MD Saunders BP Wilkinson KH Most dysplasia in ulcerative colitis is visible at colonoscopy. Gastrointest Endosc 2004;60:334–9 15332019 10.1016/s0016-5107(04)01710-9

[48] Kara MA Peters FP Fockens P Endoscopic video-autofluorescence imaging followed by narrow band imaging for detecting early neoplasia in Barrett’s esophagus. Gastrointest Endosc 2006;64:176–85 16860064 10.1016/j.gie.2005.11.050

[49] Hurlstone DP Kiesslich R Thomson M Confocal chromoscopic endomicroscopy is superior to chromoscopy alone for the detection and characterisation of intraepithelial neoplasia in chronic ulcerative colitis. Gut 2008;57:196–204 18192453 10.1136/gut.2007.131359

